# A comparison of tarsal morphology and traction force in the two burying beetles *Nicrophorus nepalensis* and *Nicrophorus vespilloides* (Coleoptera, Silphidae)

**DOI:** 10.3762/bjnano.10.5

**Published:** 2019-01-04

**Authors:** Liesa Schnee, Benjamin Sampalla, Josef K Müller, Oliver Betz

**Affiliations:** 1 Institut für Evolution und Ökologie, Evolutionsbiologie der Invertebraten, Universität Tübingen, Auf der Morgenstelle 28E, 72076 Tübingen, Germany; 2 Institut für Biologie I, Evolutionsbiologie & Ökologie, Albert-Ludwigs-Universität Freiburg, Hauptstr.1, 79104 Freiburg, Germany

**Keywords:** adhesion, friction, Insecta, locomotion, tarsus

## Abstract

Our aim was to compare friction and traction forces between two burying beetle species of the genus *Nicrophorus* exhibiting different attachment abilities during climbing. Specifically, the interaction of adhesive hairs and claws during attachment with respect to various surface properties was investigated by using a 2 × 3 experimental design. Traction force was measured for two different surface energies (hydrophilic vs hydrophobic) varying in roughness from smooth to micro-rough to rough. Nanotribometric tests on single legs were also performed. The external morphology of the attachment devices investigated by scanning electron microscopy suggested higher intra-specific (intersexual) than inter-specific differences. Whereas differences between the two species in traction force were high on smooth surfaces, no differences could be detected between males and females within each species. With claws intact, both species showed the highest forces on rough surfaces, although *N. nepalensis* with clipped claws performed best on a smooth surface. However, *N. nepalensis* beetles outperformed *N. vespilloides*, which showed no differences between smooth and rough surfaces with clipped claws. Both species demonstrated poor traction forces on micro-rough surfaces. Results concerning the impact of surface polarity were inconclusive, whereas roughness more strongly affected the attachment performance in both species. Nanotribometric analyses of the fore tarsi performed on micro-rough and rough surfaces revealed higher friction in the proximal (pull) direction compared with the distal (push) direction. In these experiments, we detected neither differences in friction performance between the two species, nor clear trends concerning the influence of surface polarity. We conclude that the investigated morphological traits are not critical for the observed interspecific difference in attachment ability on smooth surfaces. Furthermore, interspecific differences in performance are only clear on smooth surfaces and vanish on micro-rough and rough surfaces. Our results suggest that even subtle differences in the adhesion-mediating secretion in closely related species might result in qualitative performance shifts.

## Introduction

Although mostly ground dwelling [1], burying beetles (Silphidae) of the genus *Nicrophorus* have recently awakened the interest of scientists in the field of bioadhesion with regard to their tarsal secretion [2–3]. So far, the measurement of the physical strength and the description of the morphological traits of the attachment devices of various insects in the context of insect adhesion have been performed with ‘good plant climbers’ [4–7]. Although burying beetles can be observed climbing plants to reach a better position from which to start flying to their carrion resources [1], they do not primarily use their tarsi in the context of plant climbing. Instead, they are specialized in the burying of carrion and thus need to be able to cling firmly onto the skin and fur of their (mammal) carrion while tunnelling under its body. In our study, the attachment performance of animals having all tarsi attached to the ground has been quantified by means of traction force measurements of entire animals, whereas the performance of single fore tarsi has been measured with a nanotribometer. Both the number and the special morphology of the tarsal tenent hairs of the fore, middle and hind tarsi have been investigated by scanning electron microscopy (SEM).

The impulse for this study was our observed difference in the climbing ability of the two congeneric burying beetle species *Nicrophorus nepalensis* Hope 1831 and *Nicrophorus vespilloides* Herbst 1783 (Coleoptera, Silphidae). Because *N. vespilloides* beetles are, in contrast to *N. nepalensis*, unable to climb up smooth vertical (glass) surfaces, the question arose regarding the functional reason for this observed difference in attachment performance. Such large performance differences between closely related insect species of the same genus have seldom been reported [6] but have the potential to provide major clues concerning the mechanisms behind insect attachment. Although burying beetles appear not to be especially adapted to smooth and slippery plant surfaces, *N. nepalensis* is known as a ‘good climber’ [2] and both the investigated species exhibit, like other burying beetles [8], many tarsal adhesive hairs. The precise way in which the viscosity of the adhesion-mediating tarsal secretion of insects influences friction performance remains the subject of debate. Nevertheless, subtle differences in the hydrocarbon profiles of the tarsal secretion, probably leading to a decreased fluidity in *N. nepalensis*, have recently been proposed as a potential reason for these observed differences, especially on smooth surfaces [3].

Surface roughness is known to affect attachment performance in insects, spiders and geckos [9–14], whereby often so-called critical roughness values have been reported that significantly reduce attachment, especially in hairy systems. The extent of the reduction in attachment success depends on the ratio between the size of the surface irregularities and that of both the claws and the adhesive hair tips; this ratio affects the close interlocking of the respective surfaces [15–17]. Another property that influences insect adhesion is surface polarity, which can be affected by surface roughness, apparently reinforcing hydrophobic or hydrophilic surface characteristics. The testing of both of these surface properties in combination in our experiments has made it possible to attain a more comprehensive view of the attachment capabilities of these beetles, which, according to their lifestyle, experience a broad variety of natural substrates ranging from soil, fur and carrion to plant surfaces (including fallen leaves).

Interestingly, within insect species, males and females can show different attachment performances attributable to sex-specific adhesive hair morphologies [10]. In rosemary beetles (*Chrysolina americana*, Chrysomelidae), only males exhibit setae with discoid terminal ends and are thus able to generate higher attachment forces on various surfaces compared with their female counterparts [18]. In the present study, we have evaluated whether similar sex-specific differences in tenent seta number and morphology also exist in *Nicrophorus* and whether this might result in corresponding differences in attachment performance. This evaluation should help in elucidating the extent to which sexual selection might have been involved in the evolutionary formation of this attachment system.

With respect to our study species and the applied methods, the following major hypotheses are addressed: (1) The number of adhesive hairs and the special morphology found in both *Nicrophorus* species is correlated with possible differences in the attachment performance between the two species and between both sexes within each species. (2) The attachment performance of both the species is affected by both surface polarity and roughness. (3) The claws influence the attachment performance only on (micro-)rough surfaces, but not on smooth ones, where they are not able to interact with any surface protuberances. (4) Single tarsi show an anisotropic frictional directionality, resulting in higher friction coefficients in the pull (compared with the push) direction. This behaviour is related to the arrangement angle of the tenent hairs on the tarsal surface.

The traction force of entire animals and the friction force of single tarsi were tested on smooth, micro-rough and rough surfaces. All the used polymer surfaces were tested in duplicate, i.e., being either hydrophilic (treated with plasma) or hydrophobic (treated with antispread). The data were compared between and within the species. The within-species comparisons were conducted between the different surface properties such as roughness and wettability.

## Results

### Tarsus morphology

**Figure 1:**
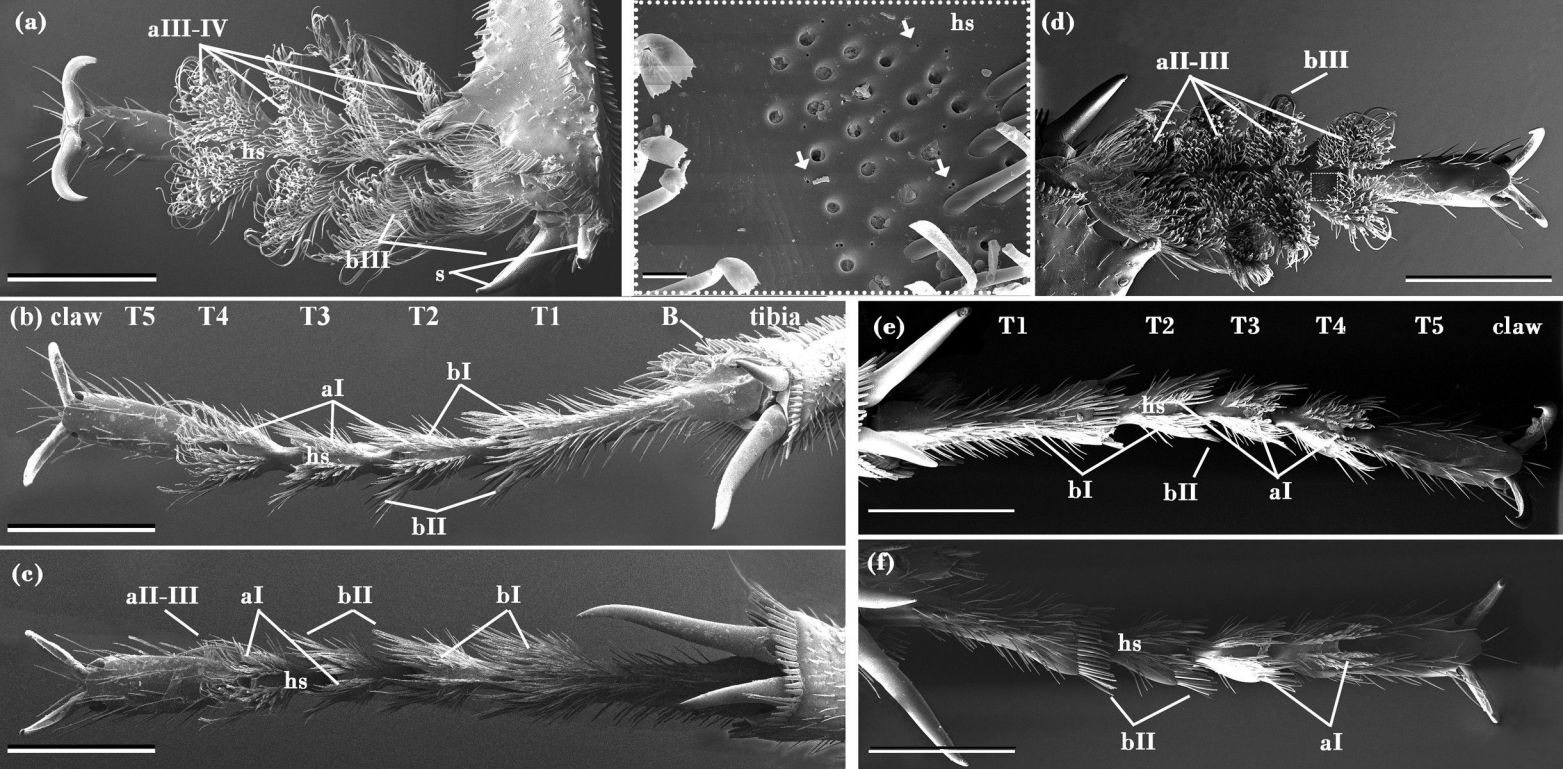
SEM images of fore (a, d), middle (b, e) and hind (c, f) tarsi of male *N. nepalensis* (a, b, c) and *N. vespilloides* (d, e, f). The tarsi are composed of five tarsomeres and a pair of hinged claws. The shovel-like widened tibia is lined with robust bristle like hairs (B) and features two prominent spines (s). The hairy pads featuring various hair types (aI–aIV and bI–bII, for their description see text and Figure 2) are divided by a hairless stripe. The inset between (a) and (d) shows pores between the hairs. The position of the inset on the tarsus is shown in (d). Scale bar: 1 mm. Scale bar (top middle), 20 µm. Abbreviations: aI–aIV and bI–bII refer to the seta types shown in Figure 2; b, robust bristle like hair; hs, hairless stripe; s, spines; T1–T5, tarsomeres 1–5.

**Figure 2:**
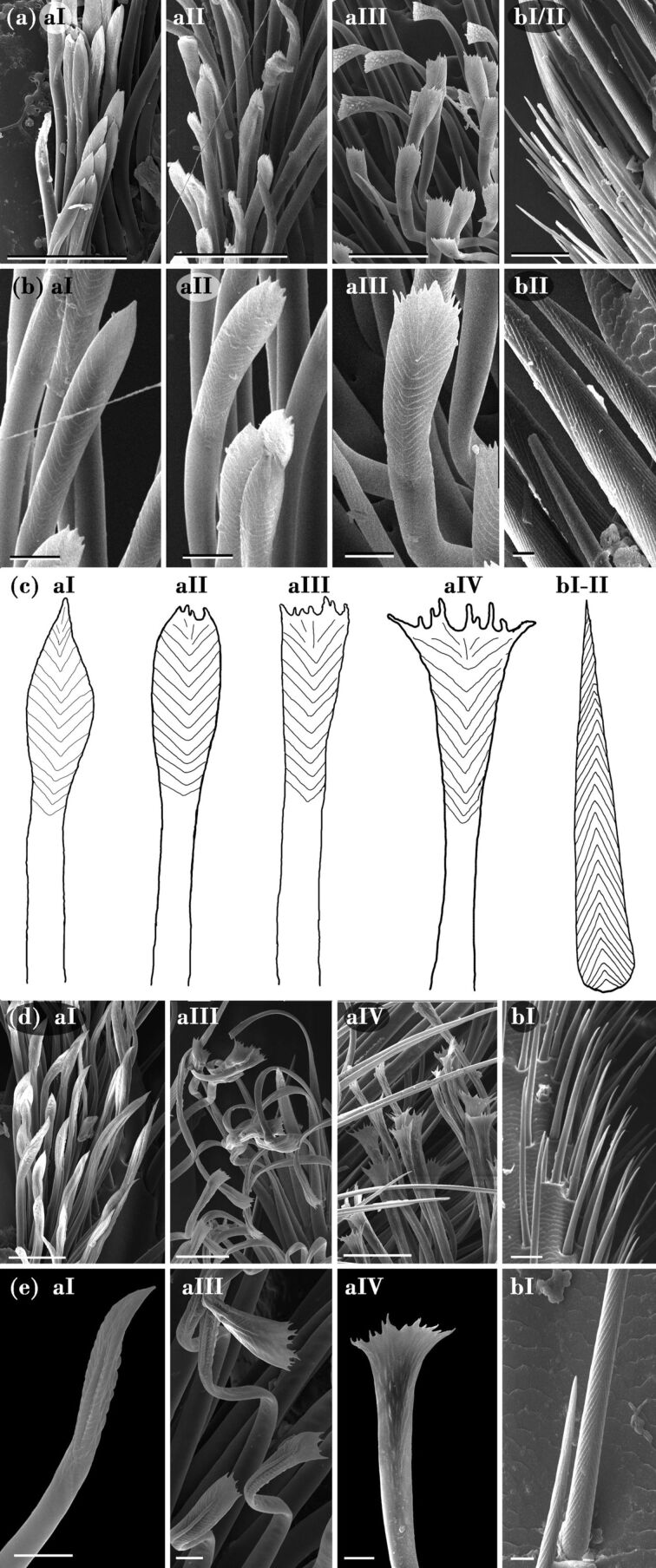
SEM images and schematic diagrams of the various types of tarsal setae found in the two *Nicrophorus* species. The abbreviations refer to the various seta types: aI, lanceolate; aII, intermediate spatula-shaped; aIII, spatula-shaped; aIV, broadly spatula-shaped and bI–II, bristle-like robust hairs. (a, b) *N. vespilloides*, (d, e) *N. nepalensis*. (c) Diagrams of the ventral side of the various tarsal seta types with hair tips having a fish-bone-like fine-ribbed structure. Scale bars in (a) and (d): 50 µm; in (b) and (e): 10 µm. For the position of the various hair types on the entire tarsus, see Figure 1.

At its distal end, the tibia of all three legs of both the *Nicrophorus* species is widened in a shovel-like manner and lined by robust bristles and two prominent spines (Figure 1a: s).The tarsi consist of five tarsomeres. The fore tarsi of *N. nepalensis* are 3357 ± 499 µm long (arithmetic mean ± sd, four males and four females were measured without claws) and vary between 324 ± 17 and 780 ± 126 µm in width depending on the tarsomere. The middle and hind tarsi are longer (4403 ± 342 µm and 4390 ± 266 µm, respectively) and narrower (min. 372 ± 15 µm on T5 hind tarsi and max. 435 ± 51 µm on T1 on middle tarsi). The smaller *N. vespilloides* shows a similar pattern (fore tarsus length: 2472 ± 339 µm, width: 320 ± 29 to 645 ± 136 µm, middle and hind tarsi length: 3763 ± 445 and 3768 ± 493 µm, width: min. 343 ± 45 µm T4 and max. 425 ± 45 µm T1 hind tarsi; measured in four males and four females). In *N. vespilloides* beetles, we found tiny pores on the ventral cuticle between the adhesive setae (Figure 1, inset: white arrows). Such pores could not be detected in *N. nepalensis*. A pair of flexibly hinged claws was seen to insert at the distal end of the fifth tarsus. The ventral side of the T1-T4 mainly bore two different types of seta. The first type were setae with distinctly broadened tips (Figure 2, aI–aIV). The second type were bristle-like hairs with pointed tips (Figure 2 bI–bII). In both species, the number of hairs with broadened tips decreased from the fore to the hind tarsus, whereby the number of bristle-like hairs was highest on the hind tarsus and lowest on the middle and fore tarsus (Table 1). The ventral hairy surfaces of the tarsi were divided by a median longitudinal hairless stripe (Figure 1: hs).

**Table 1:** Arithmetic mean number of hairs ± s.d. (sample of four males and four females), separately for type a (adhesive hairs) and type b (bristle-like hairs) for fore, middle and hind tarsi. Both the total numbers and the size-corrected numbers related to tarsus length are shown. Only hairy tarsomeres one to four (T1–T4) were taken into account, because the fifth tarsomer with the claws bears only a few bristle-like hairs.

	*N. nepalensis*					*N. vespilloides*					
	type a			type b			type a			type b	
						
	total	√#a/length		total	√#a/length		total	√#a/length		total	√#a/length

fore tarsi	1081 ± 127	10.0 ± 1.5		247 ± 69	4.5 ± 0.6		1170 ± 288	13.8 ± 0.8		220 ± 75	5.9 ± 0.6
middle tarsi	478 ± 42	5.0 ± 0.5		311 ± 36	4.0 ± 0.3		357 ± 102	5.0 ± 0.7		191 ± 40	3.7 ± 0.3
hind tarsi	291 ± 24	3.9 ± 0.3		435 ± 31	4.8 ± 0.3		173 ± 31	3.5 ± 0.4		229 ± 35	4.0 ± 0.5

The tips of hairs with broadened tips range from lanceolate to spatula-shaped and can therefore be subdivided into four classes, i.e., (1) lanceolate (aI) with a broadened plate tapering towards one apical tip (Figure 2a–e: aI), followed by (2) intermediate spatula-shaped aII ending with 4–8 apical tips (Figure 2a–c: aII), (3) spatula-shaped with a broad plate ending in 8–12 irregularly shaped apical tips (Figure 2a–e: aIII) and (4) broadly spatula-shaped only found in *N. nepalensis* (Figure 2c–e: aIV). On their dorsal side, the hair tips show a nodule-like fine structure (Figure 2e: aIV), whereas on the ventral side, a fish-bone-like fine-ribbed structure can be observed (Figure 2b: aIII, c) covering lengths between 30 and 90 µm, depending on type and location.

The total number of setae with broadened tips differs between the two species (Table 1). Whereas the total number of setae in *N. nepalensis* amounts to 1850 ± 143 on all three legs on one side, we have found 1700 ± 412 setae in *N. vespilloides*. The body-size-corrected numbers are almost identical in both species.

### Surface roughness and contact angle measurements

**Figure 3:**
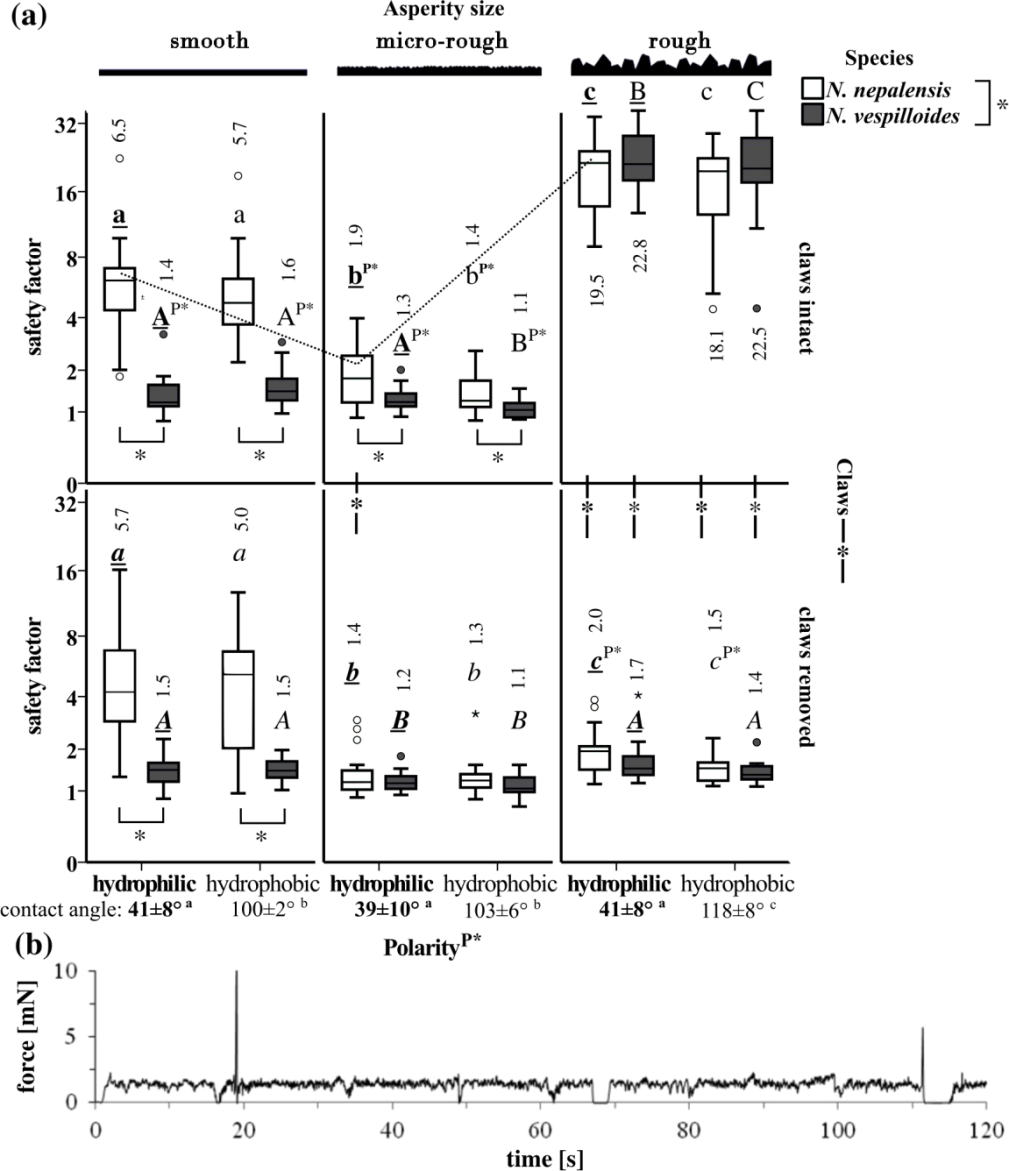
(a) Safety factors (i.e., traction force divided by weight) of the measured traction forces of both beetle species, depending on surface roughness, surface polarity and claw treatment (*N* = 20). Boxplots and the arithmetic means above or below each boxplot are shown. Results of the statistical analysis (linear mixed model with repeated measurements and paired and unpaired post hoc t-tests with Bonferroni corrected p-values, *N* = 18–20) are based on ln-transformed data. The asterisks below two boxplots indicate significant differences (*p* < 0.05) between *N. nepalensis* (white) and *N. vespilloides* (grey). Lowercase letters are used for *N. nepalensis* and uppercase letters for *N. vespilloides*. Only letters of the same format within the same species, polarity regime (statistical differences between hydrophilic and hydrophobic surfaces are indicated with P*) and claw treatment (statistical differences indicated with -*-) should be compared between the three different roughnesses (for example, see dotted line). The measured static contact angles of water (arithmetic mean ± s.d, *n* = 10) are presented below; different letters indicate statistical differences of *p* < 0.05). (b) Exemplary force-vs-time curve for a traction-force measurement of *N. vespilloides* on a micro-rough surface. The two peaks at 20 and 112 s are artefacts.

The contact angles (CA) of static droplets of doubly distilled water on the epoxy casts (10 surfaces for each combination) were about 40° on the hydrophilic surface, whereas the CA reached between 100° and 120° on the hydrophobic surface (for exact values see Figure 3). The two factor analysis of variance confirmed the significant difference between hydrophilic and hydrophobic surfaces (two-way ANOVA: surface roughness *F* = 8.5; *p* = 0.001; surface polarity *F* = 1178.6; *p* < 0.001; interaction surface roughness X surface polarity *F* = 9.4; *p* < 0.001). The post hoc *t*-test revealed no difference between the smooth, micro-rough and rough surfaces within the hydrophilic treatment. However, the CA within the hydrophobic treatment on the rough surface were significantly higher compared with those on the smooth (difference in mean value = 18.5; *p* < 0.001) and the micro-rough (difference in mean value = 15.4; *p* < 0.001) surfaces.

For the nanotribometer experiments, we used the same silanized Al_2_O_3_ grinding disc surfaces as previously described by Betz and co-workers [19]. The roughness parameters of the micro-rough and the rough Al_2_O_3_ grinding discs were as determined by Betz et al. (Table 1 in [19]) and amounted to 0.05 µm (“nano-rough”), 3 µm (“micro-rough”) and 11 µm (“rough”) (given as *R*_a_ values). These surfaces were better wettable with non-polar substances (such as diiodomethane) than with polar substances. However, in contrast to both the other surfaces, the surface with 11 µm roughness showed a higher surface polarity and an improved wettability towards polar liquids such as water. As surface free energies of real solids are always influenced by surface texture, the calculated surface free energies of 33.3 mN·m^−1^ (micro-rough surface) and 42.2 mN·m^−1^ (rough surface) (Table 2 in [19]), respectively, should not be considered as absolute values, which can only be determined for ideal, perfectly smooth surfaces [20].

The plasma-treated Al_2_O_3_ grinding disc surfaces with roughness values of 3 µm and 11 µm were not characterized by drop-shape analyses, as single droplets of doubly distilled water spread instantly over the treated surfaces, indicating a much higher surface polarity than the respective untreated surfaces.

### Traction force of animals having all tarsi attached to the ground

The mean traction force (*F*_tf_) values of animals with intact claws ranged from 1.5 mN on smooth and micro-rough surfaces to 13 mN on smooth and 45 mN on rough surfaces, depending on the species. The mean corresponding safety factors (*F*_tf_/*F*_n_) reached values between 1 and 23 (Figure 3a). During the experiments, *N. vespilloides* was in hard-running mode without any additional stimulus, which resulted in easy to read force graphs (Figure 3b). This was in contrast to the *N. nepalensis* beetles, which needed additional mechanical stimuli to keep them walking. On all the surfaces, except for the micro-rough one, these beetles tended to hold onto the surface with all six legs and pulled until they lost contact and landed on their backs. In *N. vespilloides*, such behaviour was observed on the rough (30 µm) surface with claws intact only, whereas on the smooth and the micro-rough surfaces, they lost foothold repeatedly. The resulting movement pattern resembled walking on the spot.

The linear mixed model (LMM) with repeated measurements revealed that all the tested factors except sex had a significant effect on the observed “safety factors” (in our case defined as traction force divided by body weight) (Table 2).

**Table 2:** Results of global traction force experiments. Linear mixed model, with repeated measurements of the safety factor regarding species, sex, surface roughness and surface polarity as main factors, and their interaction terms.^a^

source of variation	numerator d.f.	denominator d.f.	*F* value	significance

constant term	1	36.1	576.9	<0.0001
species	1	36.1	31.8	<0.0001
sex	1	36.1	0.1	0.730
claws	1	412.1	965.0	<0.0001
roughness	2	412.1	858.9	<0.0001
polarity	1	412.1	11.5	0.001
roughness * polarity	2	412.2	1.8	0.173
claws * roughness	2	412.2	770.7	<0.0001
species * roughness	2	412.1	172.3	<0.0001
species * claws * roughness	3	412.2	5.7	0.001

^a^d.f., degrees of freedom; *F*, test statistic; dependent variable: ln(safety factor).

### Difference between the species

The difference between the two species depended on the surface roughness and the interaction between the surface roughness and the claw treatment. The most obvious difference between the two species was observed on the smooth surface with and without claws (Figure 3). A small (still significant) difference between the species was also detected on the micro-rough surface with intact claws. With claws removed, no differences were seen on this surface. On the rough surface, *N. vespilloides* and *N. nepalensis* exhibited no differences with respect to their traction force, either with intact or with removed claws.

#### Effect of claw treatment and surface roughness

Both claw treatment and surface roughness had the highest impact on the traction force followed by their interaction term (Table 2). Both species reached their highest safety factors on the rough surface with intact claws and the lowest on the micro-rough surface with removed claws (Figure 3a). The removal of the claw tips resulted in a remarkable reduction in the safety factors (from on average of ca. 21 to about 1.7) on the rough surface, whereas no such effect was observed on the other two test surfaces (except *N. nepalensis* on the micro-rough hydrophilic surface).

#### Effect of wettability of test surfaces

The smallest but still significant impact on the safety factor was the factor surface polarity. However, the pairwise comparison for both species and the three tested surface roughnesses revealed that this effect was not consistent across all the surfaces. In general, the beetles of both species showed slightly higher attachment abilities on hydrophilic surfaces compared with values on hydrophobic ones, independent of the surface roughness (cf. Table 2: interaction between surface roughness and polarity). The only exception was for *N. vespilloides* on smooth surfaces with claws intact (see Figure 3a, upper left part). In this case, the traction force was slightly increased on the hydrophobic surface.

#### Nanotribometric measurements of friction of single fore tarsi

**Figure 4:**
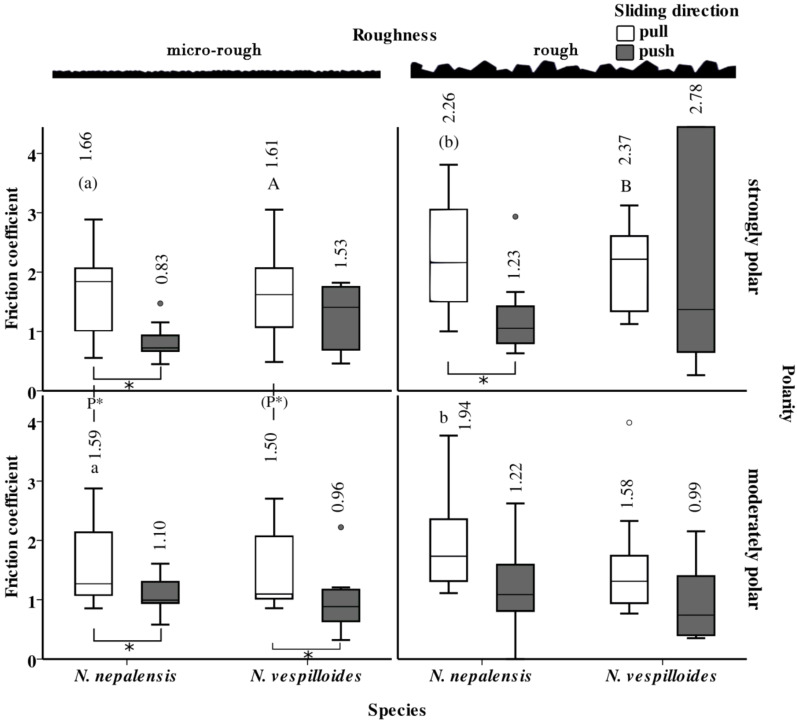
Sliding friction performance of the fore tarsus of the two *Nicrophorus* species as established during nanotribometer measurements with respect to two different surface roughnesses and polarities. Boxplots and the exact arithmetic means above each bar are shown. Different letters above or beside the bars are indicative of significant differences (*p* < 0.05, paired *t*-tests, *N* = 7–10) between the different surface roughnesses or polarities under otherwise identical conditions. Letters in parentheses indicate statistical differences by trend (*p* < 0.1). Normal font lowercase letters are used for *N. nepalensis*, normal font uppercase letters for *N. vespilloides*. Only letters of the same format within the same species, polarity regime and sliding direction (i.e., push or pull) should be compared between the two different roughnesses. P* is used to show significant differences attributable to polarity within the same species, surface roughness and sliding direction. Different sliding directions are indicated by the different colours of the boxplots: white: proximad (pull), grey: distad (push). The asterisks below two bars, respectively, are indicative of significant differences between the pull (white) and the push (grey) direction (*p* < 0.05).

Because of the overall correspondence between the pattern of the static and the sliding friction, we focus, in the following, on the sliding friction coefficient only. Whereas no difference in friction performance was apparent between the two species (Figure 4), the LMM for repeated measures revealed a significant influence of the sliding direction, the surface roughness and the surface polarity (Table 3).

**Table 3:** Result of nanotribometer experiments. Linear mixed model for repeated measurements of the sliding friction coefficient of the fore tarsus, with regard to species, surface roughness, surface polarity and sliding direction as main factors, and their interaction terms.^a^

source of variation	numerator d.f.	denominator d.f.	*F* value	significance

species	1	103.6	0.2	0.663
roughness	1	103.6	5.1	0.026
polarity	1	103.6	4.8	0.031
direction	1	103.6	17.8	<0.0001
species * polarity	1	103.6	4.7	0.032

^a^d.f., degrees of freedom; *F*, test statistic; dependent variable: ln(friction coefficient + 1).

We subsequently conducted pairwise comparisons between both sliding directions (push vs pull), the two surface roughnesses (micro-rough vs rough) and the two polarities (strongly polar vs moderately polar), keeping all the other factors constant (Figure 4). In almost all cases, friction was higher in the proximal (pull) direction compared with the distal (push) direction (for significant differences, see Figure 4). The influence of surface roughness was only detectable in the pull direction and not in the push direction. Usually, it was increased on the rough relative to the micro-rough surface. Polarity effects were only observed in the pull direction on the micro-rough surface, whereby friction was slightly increased on the polar surface.

## Discussion

The present study is a comparison of the attachment performance between two closely related beetle species. It was initiated with regard to our previous observation that *N. nepalensis* beetles can strongly attach to smooth surfaces, whereas *N. vespilloides* cannot. This finding led us to inquire into the potential mechanisms responsible for generating major differences in insect attachment performance between closely related species. Our results suggest that, on smooth surfaces, even subtle differences in the adhesion-mediating secretion might be responsible for the observed general difference between the two species in their ability to attach to smooth surfaces. Several experiments were conducted to determine various aspects of the attachment system consisting of hairs and claws.

### Tarsus morphology

Both species show a five-segmented tarsus, distally with a hinged paired claw as described for the burying beetle *N. humator* [8]. At each tibia, the beetles possess prominent spines (Figure 1a). Such spines and claws are not only important for climbing cylindrical rods, but also for locomotion in soil [21–22]. These findings correspond to the ground digging behaviour of burying beetles, and their observed behaviour of climbing blades of grass to achieve a better starting position for flight [1].

Our SEM studies have revealed a sexual dimorphism in both the number and the morphology of the tarsal adhesive setae. As reported for other burying beetles and, for example, various leaf beetle species [10,18], we have found bristle-like hairs with pointed tips (type b, cf. Figure 2). Because of their broadened tips, the type-a hairs are assumed to function mainly as adhesive hairs, whereby they might support attachment to the fur and skin of their carrion. The back of all adhesive hairs are covered by nodule-like structures (Figure 2: SEM image of aIV), as previously described for *N. humator* and other beetles such as *Cicindela campestris* (Coleoptera, Cicindelinae) [8]. Such structures may prevent agglutination of hairs [23] and might be of special importance in a moist soil environment. In general, males seem to show adhesive hair tips with broader endings compared with those of the females. Especially in *N. nepalensis*, the differences on the fore tarsi are notable, since the spatula-shaped type-aIV hairs dominate in males, whereas females predominantly show the lanceolate type aI. Although the type of adhesive setae varies greatly between males and females, no sex-dependent difference in traction force has been seen on either combination of surface structure. However, the established morphological differences might take effect only under certain biological contexts such as mating or parental care, which were not measured in our test setup. Measurements on the Colorado potato beetle *Leptinotarsa decemlineata* (Coleoptera: Chrysomelidae), which is known for its sexual dimorphism in adhesive hairs, have shown higher attachment forces for males on smooth surfaces and for females on rough surfaces. In this species, males possess additional hairs with discoidal terminal plates, featuring a completely different action mode for adhesion than the spatula-shaped setae [10]. Furthermore, artificial models of these discoidal setae are known to reach remarkable force values on smooth surfaces [24]. However, in both the *Nicrophorus* species, the established differences in hair types between the sexes are not as fundamental, especially since the different types only vary in their width.

Independent of sex, all adhesive setae are generally more elongated towards the distal end of the tarsomere, which seems to be a general principle in insects and spiders [8,11,25–26]. As assumed in [10], distal setae are essential for initiating surface contact and are therefore longer and more differentiated, as described for many other beetle species [8,25]. In terms of the discharge of the adhesion-mediating secretion, other staphylinoids such as *Philonthus* and *Stenus* (Coleoptera, Staphylinidae) have a system of very fine pore canals in the wall of the setal shaft [25,27]. In *Philonthus* species, these pores are associated with a transverse (fish-bone-like) ribbed structure at the distal hair shaft; such a structure is also present in the investigated *Nicrophorus* beetles (Figure 2b–c: aI-aIV).

### Importance of claws for traction force

In our traction force experiments, claw removal generally led to an overall reduction of the traction force. With intact claws, both beetle species showed the highest traction forces on the rough surface. The claw tips, varying across both species with diameters between 8 and 12 µm, are in the range or smaller than the surface irregularities of 11 μm asperity size. Therefore, they can easily interlock with the surface asperities, leading to high attachment forces [17]. Previous studies on the effect of claws have revealed that a decrease after claw ablation is detectable on rough surfaces, but not on smooth or micro-rough surfaces [6,16,28]. *N. nepalensis* beetles showed a slight reduction in attachment force (although not always significant) on smooth and micro-rough surfaces after claw removal. While accidental cuts of the unguitractor tendon and insufficient recovery times can be excluded, a possible functional synergism between claw and tenent setae might explain this result [6].

#### Influence of surface roughness and polarity on traction force of tenent setae only

In this section, we will only discuss tarsal attachment under the condition of removed claws, i.e., only the adhesive setae are considered. In other insect attachment experiments, the highest forces without claws were in general reached on smooth surfaces compared with any kind of structured surface [14,16]. In our case, this held true for *N. nepalensis* only, whereas *N. vespilloides* did not attain any notable traction on a smooth surface (cf. Figure 3). In agreement with other attachment-force measurements on other beetles with clipped claws [16], the beetles of both species showed a decrease in traction force on micro-rough surfaces (in comparison with the smooth surface), with a drastic reduction also being visible in *N. nepalensis* on the rough surface (cf. Figure 3). Therefore, in *N. nepalensis*, micro-rough and rough surface structuring led to a considerable reduction in attachment force compared with the smooth surface, as also shown in other insects [10,29]. This is in accordance with the relatively large setal tips in insects and especially beetles, whereas such effects are less severe in spiders and geckoes [11]. In both species and in both the global traction and the nanotribometer experiments, the lowest traction forces were attained on the micro-rough surfaces (cf. Figure 3 and Figure 4) indicating that this surface represents a critical (friction reducing) surface asperity not only for the claws, but also for the tenent setae. This means that, at certain ranges of substrate roughness, attachment organs show a minimum of adhesion. Such a critical range of roughness is explained by the reduction of the contact area between small surface irregularities in relation to the size and the shape of the setal tips [10,30]. Critical surface asperities are well known to play an important role, especially in hairy attachment systems [10,14,31], whereas such effects are less relevant in smooth systems that can obviously better compensate a wide range of roughness by their pliable pad cuticle in interaction with the fluid surface film [32].

In order to separate the effect of surface polarity from structuring, tests were conducted on hydrophilized and hydrophobized epoxy resin replicas. In both the traction and the nanotribometer experiments, the linear mixed model identified surface polarity as a relatively small but still significant factor. These results are in accordance with England et al. [33] (although these authors found no significant effect of the chemical composition of the test substrate), who proposed that, especially on structured surfaces, the effect of surface polarity is reduced. As has previously been shown, the tarsal secretions in insects contain both hydrophobic and hydrophilic components [34–37] and are proposed to mediate between the attachment structures of the animal and the various surfaces in their environment [38]. In most of our experiments, both *Nicrophorus* species showed higher traction forces (although not always significant) on hydrophilic surfaces. Similar results with better attachment on hydrophilic surfaces (but not consistent for all comparisons) have been observed in the sawfly larvae of *Rhadinoceraea micans* [39] and in adult leaf beetles of *Gastrophysa viridula* [40]. Moreover, if present, the absolute differences between the different polarities in our experiments were small and depended, for the smooth and micro-rough surface, on the claw treatment. Therefore, we conclude that the wettability of the surfaces plays a minor role compared with the surface roughness; this might be attributable to the chemical composition of the adhesion-mediating secretion of the beetles. Although only its non-polar fraction has been investigated so far, polar components such as proteins/peptides and carbohydrates might contribute to its amphiphilic property.

#### Directionality of friction force

Our nanotribometer experiments (performed on the fore tarsi) clearly revealed a direction-dependent (anisotropic) friction effect, i.e., the friction forces were significantly higher (up to two-times) in the proximal (pull) compared with the distal (push) direction (cf. Figure 4). Such directionality of orientation-controlled switch between attachment and detachment with adhesion strength varying strongly with the direction of pulling is well known in the attachment systems of insects, spiders and geckos [41]. Whereas in smooth systems, directionality results from a drop in contact area attributable to the flexibility of the pad, in hairy systems, both the contact area of each single hair and the higher shear stress, i.e., the “quality” of contact, are decisive [42]. In hairy systems, this mechanism is supported by the distadly angled insertion of the hairs (in analogy to a broom), facilitating detachment during a distal push, whereas the flat angle during the proximal acts against contact peeling [28,42–43]. For theoretical access to the problem of frictional anisotropy considering factors such as the slope of the surface structures, the rigidity of the joints, and sliding speed, the reader is referred to [44].

Our results are in full accordance with previous measurements of the frictional directionality of hairy tarsal adhesive systems, such as those in leaf beetles [42] and other arthropods [45]. Interestingly, in this context, the transverse ribbing pattern at the tips of the tenent setae of type a (cf. Figure 2) is restricted to the ventral side. If this ribbing pattern is associated with the draining pores that discharge the adhesion-mediating secretion as demonstrated in the staphylind beetle *Philonthus marginatus* [27], we can assume that the secretion might only be released when the tenent setae slide in full contact with the surface during the proximal (pullig) movements. Such an asymmetric local release might support shear-induced adhesion [46] and help to dose the secretion economically. In terms of the biological role, the higher attachment force in the pull direction might help the animals to climb effectively on a variety of structures such as plants and fur and might enable the males to hold fast onto the female elytron during mating.

#### Interspecies differences in morphology and traction force

**Figure 5:**
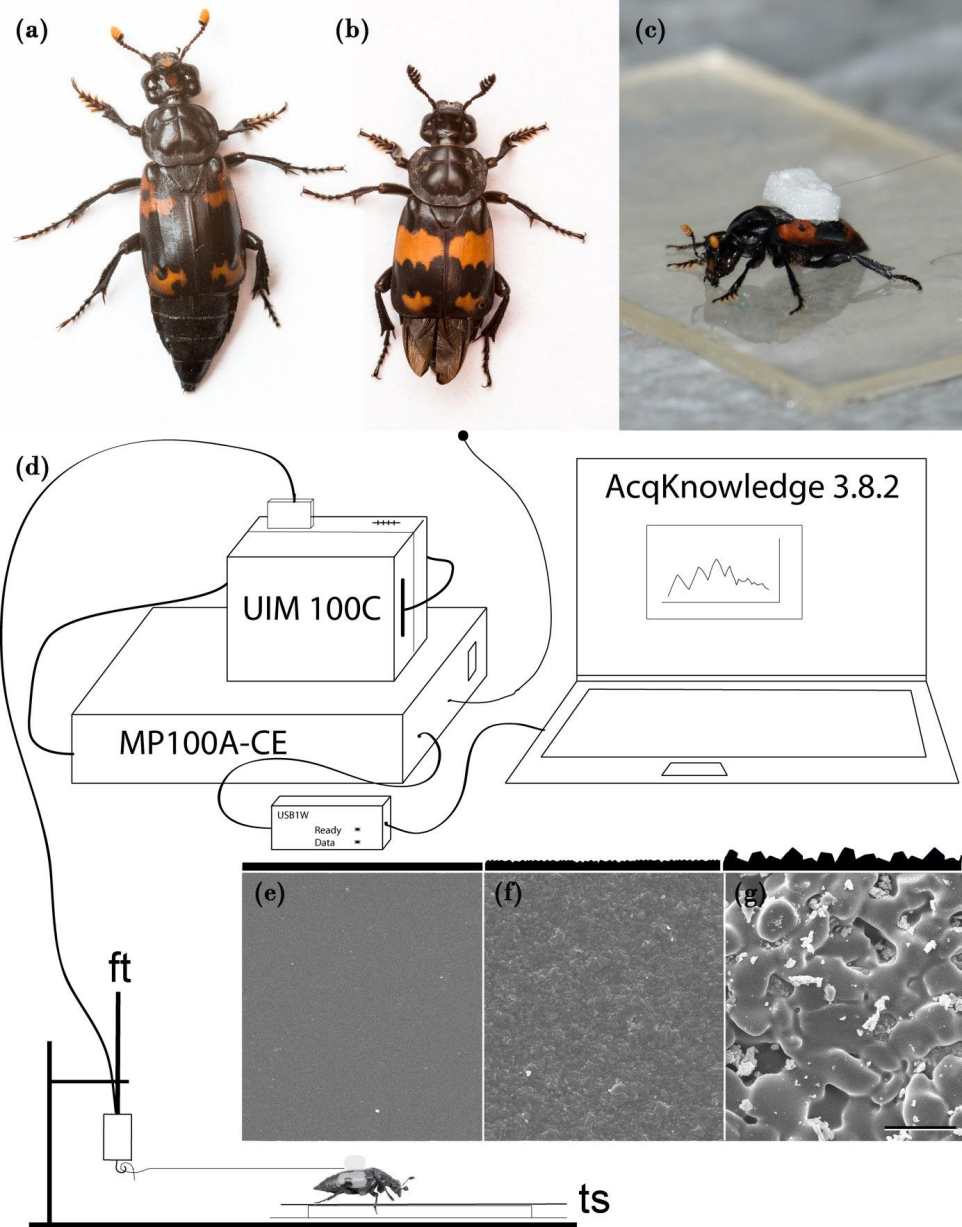
Traction-force experimental setup with the two test species, i.e., (a) *N. nepalensis* and (b) *N. vespilloides.* (c) *N. nepalensis* specimen exhibiting its maximum pulling performance on a smooth surface. (d) Experimental setup. (e–g) SEM images of the various surface structures used in the experiments (e: smooth, f: micro-rough and g: rough; scale bar: 50 µm). Abbreviations: ft, force transducer; ts, test surface.

Our traction-force experiment revealed that *N. nepalensis* attained a four-times higher traction force on the smooth surfaces than *N. vespilloides* (cf. Figure 3) supporting our previous observation that only *N. nepalensis* is able to climb vertical glass walls. However, compared with leaf beetles, and in accordance with their digging lifestyle, *Nicrophorus* beetles are not outstanding climbers on smooth surfaces. The well-studied leaf beetle *Leptinotarsa decemlineata* with a body mass between 90 and 140 mg attains traction forces between 30 and 45 mN on glass surfaces [14], whereas in our experiments, *N. vespilloides* and *N. nepalensis* (with a body mass between 250 and 400 mg) reached on average only 3 and 17 mN, respectively. Nevertheless, although *N. nepalensis* attached itself with all six tarsi to the surface and pulled (see below Figure 5c), *N. vespilloides* kept walking “on the spot”, not finding any grip. For some reason and independent of the claw treatment, these beetles are unable to attach properly to smooth surfaces. Since *N. nepalensis* beetles are larger than *N. vespilloides*, we expected them to exhibit significantly more adhesive setae, possibly explaining the observed difference in attachment performance. However, the number of hairs (both the total and the size-corrected number) does not show any notable difference that can explain this difference (Table 1). Hence, in this taxon, another means might exist to enhance the efficiency of the adhesive system on smooth systems without changing the number of hairs. As discussed previously, different types of hairs can generate different adhesive forces [47]. Whereas we have not established general differences in the morphology of the hair types (cf. Figure 2) in the two species, possible differences in the shape and the stiffness of the adhesive tips have not been investigated in this study and may play an important role [10,42]. Previous studies have suggested a decreased fluidity in the tarsal adhesion-mediating secretion in *N. nepalensis* compared with that of *N. vespilloides* [3]. Since under the dynamics of friction regimes, the generated shear stress is largely determined by the viscosity of the fluid [46], such higher viscosities might be responsible for the observed higher friction forces of *N. nepalensis* on smooth surfaces, possibly being connected to the improved yield stress and slip resistance in this species. On the other hand, under certain conditions, more viscous fluids might also be able to reduce friction (e.g., because of reduced wetting properties). Hence, our conclusion remains a matter of speculation (1) following the indirect evidence of our previous chemical analysis [3] and (2) because, in the present contribution, we have found no significant morphological or behavioural clues that can explain this contrasting attachment performance.

Notwithstanding, the established differences between the two *Nicrophorus* species suggest that, even between closely related species, the composition of the tarsal adhesion-mediating secretion can be adjusted to functional demands related to species-specific ecology. Since no robust studies are available concerning the natural history of *N. nepalenis*, the biological meaning of its increased attachment performance on smooth surfaces is a matter of speculation. Whereas *N. vespilloides* beetles are primarily soil-dwelling, *N. nepalensis* beetles might show a more vertical (arboreal) orientation during their search for carrion in the tropical forest canopies of their oriental range. This might make it necessary to be firmly attached to plant leaves. On the other hand, cuticular hydrocarbons are known to be linked to the climatic niche of an insect [48] and, thus, the assumed lower viscosity of the secretion in *N. vespilloides* might have resulted from the demand to keep it fluid under the lower environmental temperatures than those experienced by its tropical congeneric *N. nepalensis*. The higher tropical temperatures might have exerted selective pressures that have kept the cuticular lipid layer (from which the tarsal secretion is derived) more viscous to maintain its waterproofing function. In such a scenario, the increased adhesiveness of the tarsal secretion of *N. nepalensis* might have evolved as an evolutionary by-product that has brought about additional functional advantages in terms of attachment to smooth surfaces.

## Conclusion

Numerous single-species studies have successfully investigated the basic mechanisms behind insect attachment by using sophisticated test setups taken from biomechanical and materials scientific research. Complementary comparative approaches intended to correlate even subtle interspecific differences in attachment performance to structural or behavioural differences can contribute greatly to our understanding of these mechanisms. Such approaches might help to identify key factors that largely determine the reason that certain specimens attain specific performances on certain substrates and in various biological contexts such as locomotion, mating or capture of prey. Although burying beetles usually exhibit a lifestyle of ground digging, *Nicrophorus nepalensis* beetles are able to efficiently climb upon smooth surfaces such as glass, in contrast to other congeneric species such as *N. vespilloides*. Although this finding was the motivation of our research, we have conducted multiple experiments that have helped generally to evaluate the influence of surface polarity and surface roughness on the attachment capability of claws and/or tenent setae. Our micro-rough surfaces represent a critical roughness that significantly reduces the attachment capability of both the claws and the tenent setae. Whereas the claws are responsible for high attachment on the rough surface, the tenent setae are decisive on the smooth surface, but only in the case of *N. nepalensis*. Since no relevant structural or behavioural differences between these two species have been detected, we conclude that more subtle and hitherto often neglected factors might explain this phenomenon, such as the material compliance of the tenent setae and/or the chemical composition of the adhesion-mediating secretion. Although we have not investigated the compliance of the setal tips, we have found in a previous study [3] clues that the secretion of *N. nepalensis* is less fluid (i.e., more viscous). This suggests that even slight differences in the chemistry of adhesion-mediating secretions in closely related species can result in qualitative performance shifts, determining their use of certain structures of the habitat. In *Nicrophorus* species (and other insects), subtle changes of the chemical composition of the cuticular surface secretion might be metabolically easy to achieve. Selection pressures other than locomotion (e.g., climatic conditions) [2] might have been involved but might have simultaneously resulted in species such as *N. nepalensis* becoming more arboreal.

Tarsal insect attachment usually implies directionality, i.e., the friction forces depend on the sliding direction, which is a major feature ensuring controllability, i.e., the ability of the tarsi to rapidly attach to and detach from a variety of surfaces. Whereas such behaviour has mainly been demonstrated for smooth tarsal attachment systems [19,49–50], little such evidence exists for hairy systems [51–53]. As expected, in both the investigated *Nicrophorus* species, the friction forces of the fore tarsi increase in the pull direction corresponding to the alignment and the apical structure of the tenent hairs. Clarification needs to be obtained regarding the way that the fore, the middle and the hind tarsi are employed during natural locomotion at different inclinations and how this corresponds to the generation of forces in the pull and push directions.

## Experimental

### Study animals

The animals studied are two representatives of the genus *Nicrophorus* (Coleoptera, Silphidae), i.e., *Nicrophorus nepalensis* Hope 1831 (Figure 5a) and *N. vespilloides* Herbst 1783 (Figure 5b). All individuals were laboratory-reared and kept in small groups (3–5 individuals) in plastic containers (10 cm × 10 cm × 6.5 cm), filled to two-thirds of the volume with humid soil, in a climate cabinet (day: increase up to 20 °C, night: 13 °C, day/night: 12 h/12 h). Twice a week, they were fed with a beheaded yellow mealworm. For experiments, individuals were cleaned and separated for identification during experiments by being housed in small plastic containers (diameter: 5 cm, height: 8 cm) covered with a humid paper towel, which was daily replaced several times over the three-day test period. The body masses amounted to 204 ± 44 mg in *N. vespilloides* (males: 212 ± 46 mg, females: 196 ± 41 mg) and 279 ± 41 mg in *N. nepalensis* (males: 276 ± 35 mg, females: 281 ± 48 mg; arithmetic means ± s.d. *n* = 10 for both males and females).

### Morphology

Scanning electron microscopy (SEM) was used to characterize the external morphology of the tarsi. Tarsi of freshly deceased beetles were cleaned (fine brush) and air-dried in a desiccator for at least one week. The tarsi were individually mounted on a modified stub and sputter-coated with gold (approximately 40 nm; EMITECH K550X). Images of both the ventral and the lateral views of the fore, middle and hind tarsi were taken (EVO LS10, ZEISS). Lengths, widths and the number of adhesive and bristle-like hairs for each tarsomere were calculated for one fore, middle and hind tarsus per individual. The exact description of the adhesive hairs followed the terminology of Stork [8]. For each species, four males and four females were examined. The diameters of some claw tips were also measured to gain further information on the effect of the claws.

### Preparation of test surfaces and contact angle measurements

#### Traction-force experiments

In order to investigate the effect of various roughness scales on insect attachment forces, traction force experiments (Figure 5c–d) were conducted on epoxy casts (Epoxydharz L^®^, No 236349, Conrad electronics, Hirschau, Deutschland) made by using a two-step-method [54], on glass slides (Figure 5e: smooth) and on Al_2_O_3_ polishing paper (261X Lapping Film, 3M Deutschland GmbH, Neuss, Germany) with nominal asperity sizes of “1 µm” (Figure 5f: micro-rough) and “30 µm” (Figure 5g: rough) (asperity sizes according to the information of the manufacturer). The associated true roughness parameters are provided in [19]. They amounted to 3 and 11 µm, respectively. Generally, the tests were conducted on hydrophilic and hydrophobic surfaces of each roughness. For hydrophilization, the epoxy casts were treated for 5 min with plasma at 30 mA and 0.2 mbar (SCD 030; Balzers Union FL-9496 Balzers, Liechtenstein). For hydrophobization, the casts were placed for 10 s in Antispread E2 (E2/200 Fluorocarbon 60, Horb-Ahldorf, Germany) solution and air-dried for 24 h at room temperature, a method used previously [14,55]. All treated surfaces were controlled by means of static contact angle measurements by using five 4 µL droplets of doubly distilled water. Images were taken and analysed with Contact Angle plugin in ImageJ [56–57]. The five values per measured test surface were averaged for each surface, with 60 surfaces being tested in total (10 test surfaces per roughness and polarity in a 2 × 3 design).

#### Nanotribometric friction measurements

For the nanotribometric friction measurements, non-polar and strongly polar Al_2_O_3_ polishing papers were made to give the two different roughnesses (micro-rough and rough). Al_2_O_3_ polishing papers were silanized for the production of non-polar test surfaces [19], whereas for polar surfaces the polishing papers were treated with plasma as for the epoxy casts described above.

#### Traction force experiments

A small piece (2 mm × 2 mm) of styrofoam with a human hair of ca. 15 cm in length was glued longitudinally to the surface of the elytron (Figure 5c). For a recovery time of 30 min, the beetles were kept on clean moist filter paper. The animals were tethered to a force transducer by using the human hair and animated to pull forward by placing a dead mouse in front of the test surface, just out of reach. If beetles did not voluntarily start pulling, they were mechanically prodded with a small paintbrush. The generated traction force was recorded over a period of 2 min. The three highest peaks, with a time gap of at least 3 s in between, were used to calculate the individual mean pulling force for each surface. Safety factors were calculated by dividing the traction force by the previously determined body weight of the individual.

To test for the effect of claws, the experiments were conducted first with intact claws and then, a second time, two days later, with the claws removed. Animals were placed in a refrigerator for up to 30 min to slow them down, so that the claws could be removed at the base with a razor blade under a binocular dissection microscope. After one day of recovery, the experiments were repeated. Each beetle was presented with one replica of each test surface in a random order. In total, the experiments were conducted with 20 individuals (10 males, 10 females) per species. Body weights were measured with a microscale (GR-202-EL, A&D Instruments Ltd, Great Britain).

#### Nanotribometric friction experiments

Apart from the testing of the influence of surface roughness and polarity, experiments were conducted to test for possible frictional anisotropies between the distal (push) and the proximal (pull) sliding directions. One day prior to the experiments, adult *N. nepalensis* and *N. vespilloides* beetles were individually kept in clean polypropylene beakers on blotting paper moistened with demineralized water. This tissue was exchanged with a new one 30 min before the start of the experiment. The beetles were anaesthetized with ethyl acetate and positioned in a self-fabricated mount made of duroplast (a thermosetting polymer). The animals were fixed by means of a combination of Parafilm (Bemis Company, Wisconsin, USA) and insect pins. The tarsi were carefully glued (UHU supergel, Bühl, Germany) so that their dorsal side was attached to a microscope slide, with their tenent setae being exposed away from the animal. The tarsi were aligned in the horizontal direction, all five tarsomeres being straightened out and immobilized. Tribological measurements were performed with the nanotribometer NTR^2^ (CSM Instruments, Peseux, Switzerland) equipped with a dual-beam cantilever STH-001 as previously described [19]. This cantilever features a highly sensitive dual-beam spring able to measure forces in the *x*-direction (*F*_t_, stiffness = 4.8139 mN/µm) and *z*-direction (*F*_n_, stiffness = 0.5122 mN/µm) with a resolution of 30 nN. Friction forces were detected by two independent high-resolution capacitive sensors, whereas a piezo actuator provided smooth and steady motion at a slow pace. The actual measuring head consisted of a metal pin, which had an aluminum oxide (Al_2_O_3_) surface at its tip (6.3 mm^2^). All experiments were carried out at room temperature (ca. 22 °C) and a relative humidity of ca. 50%. Friction was determined on the fore tarsus for five males and five females (*n* = 10). The measuring head was dragged over the surface with a load of 2 mN and a velocity of 100 µm/s. For each measurement, the measuring head moved with an oscillating motion of the measuring table of five cycles over the surface, whereby one cycle represented one full forward (distad: push) and backward (proximad: pull) motion with a distance of 100 µm in each direction. The coefficients of static and sliding friction for each half cycle were extracted by automated analysis with a script written in Python 2.7 [58] and NumPy [59]. The arithmetic means of the coefficients of friction of the three middle cycles of a measurement were taken for further statistical analyses. We recorded 12 extreme values that exceeded three boxplot lengths; these were excluded as outliers from further analyses.

### Statistics

We used the same individuals for measuring the effects of the various treatments, so that we could use test statistics for repeated measurements by comparing the different treatments. Before statistical analysis, the data were ln-transformed to better fit a normal distribution and homogenize the variances. Since, in the case of the nanotribometric measurements, values between 0 and 1 also occurred, the value 1 was added to each measurement value before logarithmization. A linear mixed model (LMM) with repeated measurements was conducted in order to analyse the importance of the various factors that possibly explained both the observed traction forces and the nanotribometric data. We used paired *t*-tests with subsequent Bonferroni correction. Whereas both Shapiro–Wilk tests (with *n* ≥ 20) and visual inspections of our data confirmed a normal distribution in the vast majority of the groups to be compared, in few cases, the Shapiro–Wilk test revealed significant rejections of the null hypothesis. However, since in these cases the data could be visually confirmed as having a near-normal distribution, we considered them as almost normal in view of the robustness of the *t*-test against slight violations of the assumption of normal distribution.

The contact angle data were also visually checked for their normal distribution. For further analysis of these data, we employed a two-way ANOVA and a pairwise *t*-test with Bonferroni-corrected *p*-values as post hoc tests. All analyses were performed in SPSS 22 [60].
